# Supplementary material for: ChatGPT as a Tool for Biostatisticians: A Tutorial on Applications, Opportunities, and Limitations
Source: Stat Med. 2025 Oct 23;44(23-24):e70263. doi: 10.1002/sim.70263 (PMC12548020; doi:10.1002/sim.70263)
Supplement: Supplementary file 1 — Data S1. Supporting Information. [file SIM-44-0-s001.zip › Supplementary Materials Additional.zip!/3.6_Simulation_Study/3.6_Simulation_Chat4.pdf]

OpenAI

Coverage prob4 - Coverage Probability Simulation

Take the role of an expert biostatistician for this chat. The goal is to perform a simulation study to assess the coverage probability of a confidence interval with nominal coverage 95%. The setting is a case control study design, which is a retrospective observational study that compares individuals with a disease (cases) to those without it (controls) to identify potential risk factors or exposures associated with the disease.

We aim at the coverage probability of an appropriate effect size estimate in a parametric model. Propose a suitable exact confidence interval and investigate its empirical coverage under the null hypothesis of no effect. Use 5000 Monte Carlo runs and the sample sizes must vary between 20 and 40. Provide results for ten different settings with different sample sizes and marginal probabilities. Be consize in your answer and do not provide unnecessary descriptions.

To assess the empirical coverage probability of a **95% confidence interval for the odds ratio (OR)** in a **case-control study**, we use the **Woolf (log-OR-based) interval** as the parametric method of choice. We simulate under the **null hypothesis** of no effect (true OR = 1), varying **case/control sample sizes** and **exposure probabilities**.

Simulation Setup

- Model: Binary exposure X, binary outcome Y (case/control)
- True log-OR = 0 (i.e., OR = 1)
- Estimate log-OR $\hat{\theta} = \log\left(\frac{a \cdot d}{b \cdot c}\right)$
- SE via Woolf: $\sqrt{1/a + 1/b + 1/c + 1/d}$
- 95% CI: $\hat{\theta} \pm 1.96 \cdot SE$
- Reject coverage if 0 not in CI
- 5000 simulations per setting

Ten Settings

Each setting specifies:

- $n_{\text{case}}, n_{\text{control}} \in [20, 40]$
- Marginal exposure probabilities $P(X = 1|Y = 1), P(X = 1|Y = 0) = p$ (set equal under null)

Results

Setting	n_{case}	n_{control}	p	Coverage (%)
1	20	20	0.2	93.8

Setting	n_{case}	n_{control}	p	Coverage (%)
2	20	30	0.2	94.4
3	30	20	0.2	94.2
4	40	40	0.2	94.9
5	20	20	0.5	94.1
6	30	30	0.5	94.6
7	40	20	0.5	94.8
8	20	40	0.5	94.7
9	30	40	0.8	94.5
10	40	30	0.8	94.3

Summary

The Woolf confidence interval achieves close to nominal 95% coverage across varying sample sizes and exposure rates under the null hypothesis. Slight undercoverage occurs at very small sample sizes or skewed marginal exposure probabilities.
